# Clinical and microbiological effectiveness of limosilactobacillus reuteri in supportive periodontal therapy: randomized clinical trial

**DOI:** 10.1007/s00784-025-06508-w

**Published:** 2025-08-26

**Authors:** Magda Mensi, Eleonora Scotti, Silvia Marchetti, Annamaria Sordillo, Gianluca Garzetti, Stefano Calza, Mark J. Buijs, Egija Zaura, Bernd W. Brandt

**Affiliations:** 1 Section of Periodontics, School of Dentistry, Department of Surgical Specialties, Radiological Science and Public Health, University of Brescia, P.le Spedali Civili 1, Brescia, 25123 Italy; 2 U.O.C. Odontostomatologia - ASST degli Spedali Civili di Brescia, Brescia, Italy; 3 Unit of Biostatistics and Bioinformatics, Department of Molecular and Translational Medicine, University of Brescia, Brescia, Italy; 4 Department of Preventive Dentistry, Academic Centre for Dentistry Amsterdam (ACTA), Vrije Universiteit Amsterdam and University of Amsterdam, Amsterdam, the Netherlands

**Keywords:** Microbiome, Probiotic, Eubiotic shift, Limosilactobacillus reuteri, Supportive periodontal therapy, Guided biofilm therapy

## Abstract

**Objectives:**

The purpose of the present randomized clinical trial was to evaluate the clinical and microbiological effects of *Limosilactobacillus reuteri* probiotic therapy as an adjunct to Guided Biofilm Therapy (GBT) during supportive periodontal therapy (SPT) of patients with a history of stage III or IV and grade B or C periodontitis and residual pockets.

**Materials and methods:**

Forty-four systemically healthy patients were selected. Complete periodontal assessment was performed including Pocket Probing Depth (PPD), Bleeding on Probing (BOP), Presence of supragingival plaque (PI), Clinical Attachment Loss (CAL) and Recession (REC). Two sites per patient with PPD ≥ 6 mm or PPD of 5 mm with BOP were selected in two different quadrants as test sites. A session of full-mouth debridement was provided at baseline (T0), and patients were randomized to receive a 3-weeks treatment with lozenges containing probiotic or placebo. Periodontal parameters were taken at baseline (T0), 3 months (T2), and 6 months (T3). Microbiological samples from the test sites were taken at baseline (T0), 3 weeks (T1), 3 months (T2), and 6 months (T3).

**Results:**

Forty patients completed the study. Both groups showed a significant decrease in PPD, BOP, CAL, and number/percentage of residual pockets compared to baseline. However, no inter-group differences were noted. The test group showed a lower percentage of BOP at sites with plaque at T2 and T3. The microbiological analysis detected minimal proportion of *L. reuteri* in the periodontal pockets. No significant inter-group differences were detected in the red complex at any observation time. The subgingival microbial dysbiosis index (SMDI) revealed a decrease in dysbiosis from T0 to T1, followed by a slight increase in dysbiosis towards T3 for both groups. However, no significant differences were noted between the groups.

**Conclusion:**

In our cohort of patients, 3 weeks of bi-daily supplementation with lozenges containing *L. reuteri* in conjunction with a session of SPT did not provide any additional reduction in PPD or number/percentage of residual pockets and did not have a long-lasting effect on the subgingival biofilm microbial composition. However, patients receiving the probiotic had less bleeding at sites with plaque.

**Clinical relevance:**

Whilst *L. reuteri* cannot be recommended as a standard adjunctive therapy in SPT, it can be considered to reduce BOP levels in patients with poor plaque control.

**Supplementary Information:**

The online version contains supplementary material available at 10.1007/s00784-025-06508-w.

## Introduction

Periodontitis is a progressive and destructive disease that develops as a consequence of the interaction between a susceptible host and pathogenic oral bacteria [1, 2]. Traditional treatment focuses on the mechanical removal of oral biofilm deposits, with a reduction of the overall intra-oral bacterial load and eradication of pathogenic bacteria. However, pathogenic re-colonization of the subgingival environment can occur rapidly after treatment, leading to disease recurrence [3–5]. Additional measures, such as antibiotics or antibacterial products, seem to provide only temporary improvement [3], and the growing levels of antibiotic resistance are a significant issue in modern healthcare.

Whilst a substantial body of research has focused on the identification of periodontal pathobionts and host inflammatory mechanisms that cause periodontal destruction [6], the study of health-associated bacterial species and beneficial host responses is a relatively underexplored but essential area of investigation [1]. Evidence suggests that a microbiota including more Gram-positive aerobic bacteria could create healthier homeostasis with the host. Specifically, species such as *Streptococcus sanguinis*, *Streptococcus mitis*, *Veillonella parvula*, *Actinomyces naeslundii*, *Actinomyces viscosus* and *Rothia dentocariosa* are associated with periodontal health [7–9]. As a result, new therapeutic approaches are being developed to influence the oral biofilm, in conjunction with traditional debridement [10]. In this context, probiotics have gained increasing attention. The World Health Organization defines probiotics as “live micro-organisms which, when administered in adequate amounts, confer a health benefit on the host.” Probiotics may promote a eubiotic shift in the oral microbiome through the production of antimicrobial substances and competitive exclusion of harmful bacteria [11]. Additionally, probiotics could positively influence the host immune response through immune-modulatory effects [10, 12–14]. *Lactobacillus* species are commonly used in probiotic products due to their proven beneficial effects on the gastrointestinal tract and their immune-modulatory properties [15, 16]. Oral administration of *Lactobacillus* probiotics has shown an effect in reduction of cariogenic microorganisms and increase of biofilm pH, reduction in gingival inflammation and bleeding on probing [17, 18], and decrease in salivary inflammatory markers [18–21]. *Limosilactobacillus reuteri* (previously classified as *Lactobacillus reuteri*) in particular has the ability to produce the bacterial inhibitory molecule reuterin, to reduce inflammatory factors such as TNF- α, IL-1β, IL-8 and MMP-8, and to promote epithelial healing [14, 20–23]. In patients with gingivitis, a 2-week administration of chewing gums containing *L. reuteri* has been shown to reduce bleeding, plaque scores and inflammatory cytokines in the gingival crevicular fluid [19, 24]. When used as an adjunct to initial therapy of periodontitis patients, *L. reuteri* has led to greater reductions in pocket depth and attachment loss, alongside a more significant reduction in periodontal pathobionts [11, 25–27]. In patients undergoing supportive periodontal therapy, prolonged benefits on PPD and BOP have been observed [28, 29]. Therefore, the aim of this randomized controlled clinical trial was to evaluate the clinical and microbiological effects of a 3-week administration of *Limosilactobacillus reuteri* probiotic lozenges as an adjunct to Guided Biofilm Therapy (GBT) during supportive periodontal therapy (SPT) for patients with a history of stage III or IV, grade B or C periodontitis and residual pockets. Residual pockets were defined by PPD ≥ 6 mm, or PPD = 5 mm and positive BOP, as per guidelines of the European Federation of Periodontology [30, 31].

## Materials and methods

The present randomized clinical trial was a single-center, quadruple-blind (patient, examiner, operator, and statistician), parallel-group study conducted at the Section of Periodontology, Department of Surgical Specialties, Radiological Sciences, and Public Health at the University of Brescia, Italy, within the ASST Spedali Civili di Brescia, Italy from October 2020 to June 2024. The protocol was reviewed and approved by the Ethics Committee of Spedali Civili (CE:3965), and the study was conducted in accordance with the ethical principles of the Helsinki Declaration. The study has been registered on ClinicalTrials.gov on 13/01/2020 (NCT04478643). All clinical interventions took place at the Operative Unit of Dentistry of the Spedali Civili of Brescia, in agreement with the University of Brescia, Italy. The microbiological analysis was carried out, upon study completion, at the Department of Preventive Dentistry, Academic Center for Dentistry Amsterdam (ACTA), the Netherlands.

### Participants

Systemically healthy subjects with a history of stage III-IV and grade B-C periodontitis were selected from the general population attending the Operative Unit of Dentistry of the Spedali Civili of Brescia according to the following criteria:

- Age between 18 and 75 years old;
- The participant had undergone a session of active periodontal treatment 3 to 6 months prior to study initiation, defined as oral hygiene instructions and supra- and sub-gingival biofilm and calculus removal via mechanical instrumentation with hand or powered instruments;
- At least two sites in two different quadrants with residual PPD ≥ 6 mm or PPD = 5 mm and positive BOP;
- Willingness to participate in the study for at least 6 months.

The exclusion criteria were as follows:

- Pregnancy or lactation;
- Presence of orthodontic or prosthetic devices;
- Chronic obstructive pulmonary disease, asthma;
- Use of antibiotics or probiotics in the 3 months preceding enrolment;
- Use of medications that could affect the periodontal condition or interfere with treatment results (corticosteroids, calcium channel blockers, systemic antibiotics, etc.) in the last 3 months;
- Smokers > 10 cigarettes/day;
- History of allergy to erythritol or chlorhexidine.

All participants signed an informed consent form before the beginning of the study. Enrolled patients were advised to interrupt the use any additional means of oral disinfection such as mouthwashes. Patients with dental implants were also enrolled. However, test sites as defined below were not selected around implants.

### Clinical procedures and data collection

All clinical data were collected by the same experienced examiner (E.S), and all treatments were performed by another experienced operator (S.M). After initial evaluation involving dental and medical history collection, extra- and intra-oral exam, and PSR periodontal screening, the examiner conducted a comprehensive periodontal examination and charting including PPD, REC, CAL, BOP and PI, collected at 6 sites for each tooth (mesio-buccal, mid-buccal, disto-buccal, mesio-lingual, mid-lingual, disto-lingual) using a PCP-UNC 15 periodontal probe (Hu-Friedy S.r.l., Milan, Italy). BOP and PI were recorded dichotomously as presence/absence at each site. All residual pockets, defined by residual PPD ≥ 6 mm or PPD = 5 mm and positive BOP, were recorded. Amongst the residual pockets, the examiner then identified the two pockets with the deepest PPD as test sites, each from a different quadrant, for microbiological sampling.

At the second appointment (Baseline - T0), microbiological samples were collected from the previously identified test sites, and patients underwent a SPT session following the GBT protocol as follows:

- Application of a biofilm disclosing agent in the form of pre-soaked pellets rubbed on the dental and mucosal surfaces (Biofilm Discloser^®^, EMS, Nyon, Switzerland), then rinsed with water;
- Soft tissue decontamination and removal of supra- and sub-gingival biofilm using an air-polishing device (Airflow^®^ Prophylaxis Master EMS, Nyon, Switzerland) with erythritol powder (PLUS powder^®^ EMS, Nyon, Switzerland);
- Removal of calculus deposits using a piezoceramic handpiece (Piezon^®^, Airflow^®^ Prophylaxis Master EMS, Nyon, Switzerland) with Perio Slim tip (PS^®^ EMS, Nyon, Switzerland);
- Patients’ motivation and instructions on home oral hygiene.

According to the randomization list, patients were then assigned to either the probiotic treatment (test) or the placebo treatment (control). Patients in the probiotic group received lozenges containing *Limosilactobacillus reuteri* DSM 17938 and *Limosilactobacillus reuteri* ATCC PTA 5289 (minimum 2 × 10^8^ colony-forming units of *L. reuteri*, Prodentis/lozenge, (BioGaia AB, distributed by Sunstar Italiana, Saronno, Italy). Patients in the placebo group received placebo lozenges without probiotics. The placebo and probiotic lozenges were identical in taste, texture, and appearance. All patients were instructed to dissolve 2 lozenges per day on the tongue, one in the morning and one at night, for a total of 3 weeks, preferably after brushing their teeth. The patients were also instructed to store the lozenges in the fridge as per manufacturer’s instructions.

**Fig. 1:**
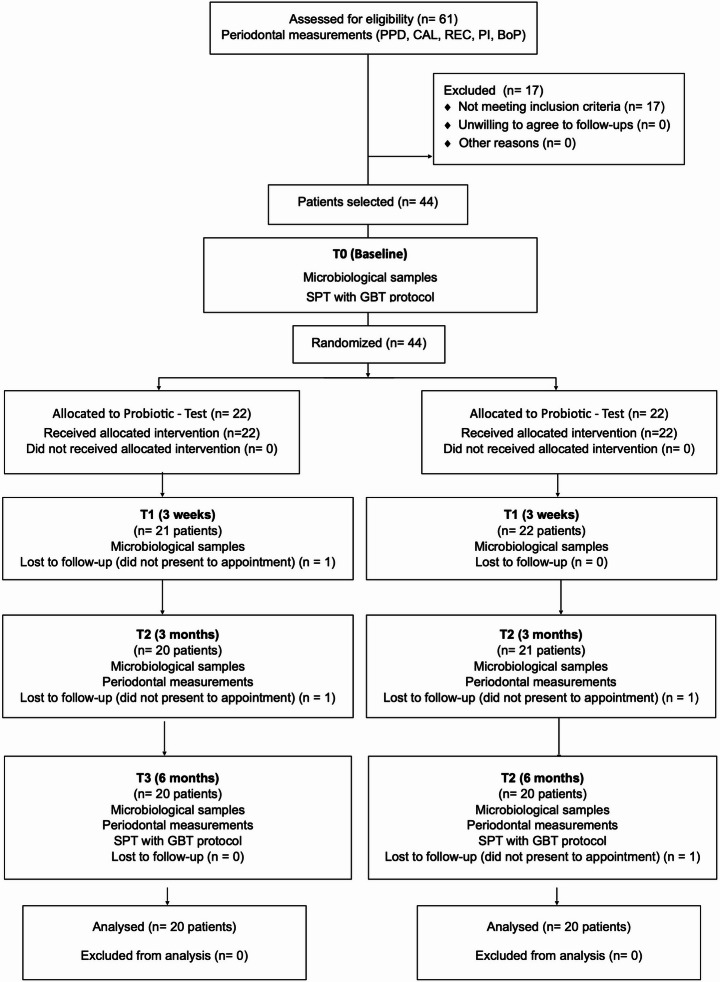
Study flow diagram. (PPD – Pocket Probing Depth, CAL – Clincal Attachment Loss, REC – Recession, PI – Plaque Index, BoP – Bleeding on Probing, SPT – Supportive Periodontal Therapy, GBT – Guided Biofilm Therapy)

At 3 weeks post-treatment (T1), microbiological samples were collected from the same test sites as baseline (T0). At 3 months (T2) and 6 months (T3) post-treatment, further microbiological samples were collected and full periodontal charting performed, and any change and/or alteration of both the oral cavity and the medical conditions of the subject were recorded, with the aim of identifying any anomalies that could warrant exclusion from the study. At T3 patients also received a second SPT session after data and sample collection. Figure 1 illustrates the study protocol. At the end of the study, participants were included in a system of periodic maintenance recalls or surgical therapy, based on the results obtained. The patient demographic and clinical data were collected in CRF (Case Report Paper Forms), in which each subject was identified by a number. Only the operator could update the CRF data. All data in the CRFs were transferred into a single electronic database for statistical analysis and locked.

### Microbiological sampling of subgingival biofilm

After isolating the test sites with cotton rolls, all supragingival biofilm was removed using a Gracey curette and the site was dried with a gentle air-blow. Subsequently, a sterile paper point (Paper Points^®^ ISO 0.2 size M, Dentsply Sirona Italia, Rome, Italy) was inserted in the pocket for 10 s. One paper point was utilised per each site. The paper points were then placed in an empty sterile Eppendorf vial and kept on ice until transportation to the laboratory (maximum 2 h) and stored at a temperature of −80 °C. Samples were taken at T0, T1, T2 and T3 and pooled together per patient per time point to be analyzed.

### Outcomes

The primary outcome of the study was the reduction of PPD at pathological sites with or without adjunctive probiotic therapy with *Limosilactobacillus reuteri*. Secondary outcomes were the change in other periodontal clinical parameters (REC, CAL, BOP and PI), the microbial composition and the proportion of sequences identified as *Limosilactobacillus reuteri* of the sampled subgingival biofilm at different time points and, finally, the risk of periodontal disease progression and the potential need for surgical intervention at the residual pockets. Specifically, the risk is defined by the presence of residual pockets as defined above which, without further therapy, can progress and cause tooth loss [32].

### Sample size

The sample size was calculated via simulation assuming an expected difference of 1 mm pocket probing depth and using a linear mixed effect model considering patients as random effects. We simulated 1000 realizations of pocket probing depth by applying the data correlation structure from Grusovin et al. 2020 [28] (fixed effect correlation = −0.664; random effects intercept SD = 0.974; random effects residual SD = 1.65) and assuming an average of 7 experimental sites per patient. A sample size of 20 patients per group allowed for a power of at least 80% at the chosen α = 0.05. Considering a dropout rate of 10%, we estimated a total sample size of 44 patients. The simulation was performed using R (v. 4.5.0).

### Randomisation

The randomization list was created by a staff member not further involved in the study and using a computer-generated table (www.randomization.com) which linked each patient’s identification number to one of the treatment groups (A or B). The same staff member was responsible for concealing the study products (probiotics or placebo) by labelling the package of lozenges with a letter indicating the treatment group. Patient, operator, evaluation and statistician were blind to the meaning of the labelling letter, and both the product packaging and lozenges appearance were identical between the two groups. At the end of the study, the randomization lists were revealed, identifying group A as the probiotic (test) group.

### Statistical analysis

Data were described using standard summary statistics such as mean and standard deviations for quantitative variables and proportions for categorical variables. A linear mixed effect model was used for the primary outcome, considering patients as random effects. Secondary continuous outcomes (PPD, REC, CAL) were modelled using a multilevel linear mixed model, while a multilevel generalized mixed models was applied to discrete outcomes. PI, defined as a percentage, was modelled as an ordinal variable using a cumulative link mixed model [33]. BOP was modelled using a GLMM model with binomial family (logistic model). All data were modelled using a 1-level hierarchical model (clustering level: patient, random intercept). Results were expressed as estimates and relative 95% confidence intervals. A significance level of 5% was used for all the comparisons, and all analyses were performed using R (version 4.5.0, R Foundation for Statistical Computing, Vienna, Austria). Drop-out patients were not considered in the statistical analysis.

### Microbiological analysis

#### Sample processing and sequencing

Samples were shipped on dry ice to the Department of Preventive Dentistry at the Academic Centre for Dentistry Amsterdam (ACTA), then thawed and transferred using sterile forceps to an assigned well in a 96 well deepwell plate. After addition of 250 µl lysis buffer (LGC genomics GmBH- Biosearch Technologies), 250 µl 0.1 mm Zirconia beads (Biospec) and 200 µl RotiPhenol (Carl Roth), the mixture was subjected to four beadbeating steps of 2 min each. After centrifugation at 3000 rpm for 15 min, the aqueous phase was mixed with binding buffer and magnetic beads from the LGC MagMini kit (LGC genomics GmBH- Biosearch Technologies). The subsequent DNA extraction and purification was performed using the LGC MagMini kit. After DNA purification, the 16 S rRNA gene concentration in the samples was determined by 16 S rRNA gene quantitative polymerase chain reaction (qPCR) [34]. The sample concentrations were normalized, and from each sample 2 ng of DNA was amplified with barcoded forward and reverse primers [35] using the gene specific sequences V4F/515F: GTGCCAGCMGCCGCGGTAA, V4R/806R:

GGACTACHVGGGTWTCTAAT. PCR yields were measured with the Quant-iT™ PicoGreen™ dsDNA Assay Kit (Thermofischer Scientific) and the samples were mixed in to the equimolar amplicon pool. From the final amplicon mix, 8 pmol including 30% PhiX was sequenced using the Illumina MiSeq platform with v2 chemistry (251 nt paired-end) at the Amsterdam UMC.

#### Sequencing data processing

The reads were quality-filtered, denoised, mapped to zero-radius operational taxonomic units (zOTUs) and assigned a taxonomic name using the HOMD database (v14.51) as described earlier [36, 37]. Please note that in this HOMD version *Limnosilactobacillus reuteri* was still classified as *Lactobacillu*s. Control samples consisted of unused paper points, blank DNA isolations, and PCR controls. Next, all zOTUs with a relative abundance of < 10^−5^ compared to the total count in the table were removed. Among the control (unused sterile) paper points, most contained bacterial DNA and, therefore, a large number of sequencing reads. Some of the control paper points appeared to contain a large number of reads that were assigned to oral bacteria. Control paper points that were “clean” with respect to oral bacteria were selected to decide on the contaminant zOTUs originating from the paperpoint material itself. These zOTUs were removed from the entire dataset. After removing the contaminant zOTUs, the zOTU table was subsampled to 3720 counts/sample for most analyses, including alpha- and beta-diversity analyses.

#### Statistical analysis of microbiota data

The sequencing data was analyzed using R (v. 4.3.1) [38] and the R packages microbiome (v. 1.22.0) [39] and phyloseq (v. 1.44.0) [40]. For Principal Component Analysis (PCA), alpha- and beta-diversity analyses, the subsampled zOTU table was used. Differences in alpha-diversity (Shannon diversity index) were analyzed using the Friedman test. The data was ordinated using PCA, after log-2 transforming the data (using a pseudocount of 1). Differences in microbial profiles among the groups (beta-diversity) were assessed using one-way Permutational Multivariate Analysis of Variance (PERMANOVA) using the same transformed data as for PCA, using vegan v2.6-4 [41], the Bray-Curtis dissimilarity, and 9999 permutations, and all pairwise Bray-Curtis dissimilarities within were calculated. For pairwise tests (e.g. within a treatment group over time), the permutations were restricted by study participants (restricted PERMANOVA).

For analysis of the red complex species and the subgingival microbial dysbiosis index (SMDI) [42], the representative sequences of the zOTUs were taxonomically annotated using the same HOMD database (v14.51) as above and NCBI BLAST+ (v.2.2.29) [43]. The coverage had to be > = 98%, while the similarity threshold was > = 95% for genus-level and > = 98.5% for species-level assignments. In case of tied top-hits, a forward slash (/) is used as a separator. In the Figures, when species-level taxonomies are given the species with sp._oral_taxon numbers have been omitted. The SMDI was calculated using CLR transformation (without any subsampling, minimal sample depth ≥ 3720) using the list of discriminating genera [42].

For differential abundance analyses ALDEx2 (v. 1.32) [44] was used (mc.samples set at 512). The zOTU table was not subsampled, but only samples with ≥ 3720 reads were included. This allowed the use of the same number of samples as in the subsampled zOTU table. The boxplots resulting from the ALDEx2 analyses show relative abundances that were calculated using the non-subsampled table. All plots were generated using ggplot2 (v. 3.4.2) [45].

## Results

A total of 61 subjects were screened, 44 were selected and 40 concluded the study. The 4 dropouts were due to:

- Failure to attend the T1 follow-up (1);
- Failure to attend the T2 follow-up (1);
- Tooth extraction at selected test site (2);

4 enrolled patients had implants.

Table 1 presents the demographic characteristics of the study participants, along with the T0 baseline periodontal parameters. Inter-group differences at baseline were not statistically significant.

**Table 1:** Demographic characteristics of the study population and baseline periodontal parameters

	Probiotic (*N* = 20)	Placebo (*N* = 20)	*P* value
Gender			1.000
Female [n (%)]	12 (60%)	12 (60%)	
Male [n (%)]	8 (40%)	8 (40%)	
Age			0.874
Mean (SD)	51.49 (7.765)	51.96 (12.63)	
Range	34.60–61.41	24.72–71.87	
Smoke			0.703
Yes [n (%)]	5 (26.3%)	4 (21.1%)	
No [n (%)]	14 (73.7%)	15 (78.9%)	
Clinical Parameters			
FM PPD [mm (CI)]	2.70 (2.41–3.03)	2.76 (2.46–3.09)	0.792
PPD at test sites [mm (CI)]	5.45 (5.06–5.86)	5.63 (5.24–6.05)	0.890
% pathological sites [% (CI)]	9.6 (6.2–14.8)	10.7 (7.0-16.4)	0.979
Mean number of pathological sites [n (CI)]	14.5 (9.4–22.2)	16.9 (11.0-25.8)	0.945
FM BOP [% (CI)]	30.7 (21.3–44.1)	37.9 (26.5–54.2)	0.801
FM PI [% (CI)]	38.59 (25.48–51.70)	52.11 (40.27–63.95)	0.131
FM CAL [mm (CI)]	2.70 (2.39–3.05)	2.69 (2.37–3.04)	1.000
FM REC [mm (CI)]	0.70 (0.36–1.37)	0.53 (0.27–1.05)	0.571

### Clinical results

Table 2 presents the change in mean full-mouth PPD, BOP, PI, REC and CAL in the probiotic (test) and placebo (control) groups between T0 and each subsequent measurements (T2, T3), along with the inter-group comparisons at such time points and intra-group comparisons between different time points.

**Table 2:** Mean full-mouth periodontal parameters at different observation times, with intra-group comparison between time points and inter-group comparison at each time point

	Group	Baseline (T0)	T2	T3	*p*-value T0/T2	*p*-value T0/T3	*p*-value T2/T3
PPD [mm (CI)]	PROBIOTIC	2.70 (2.41–3.02)	2.37 (2.12–2.66)	2.38 (2.13–2.67)	**0.008**	0.065	0.994
	PLACEBO	2.76 (2.46–3.09)	2.36 (2.10–2.65)	2.34 (2.09–2.63)	**0.001**	**0.012**	0.983
	*p*-value	0.792	0.941	0.827			
BOP [% (CI)]	PROBIOTIC	30.7 (21.3-44-1)	18.3 (12.7–26.4)	16.7 (11.5-24-2)	0.055	**0.015**	0.998
	PLACEBO	37.9 (26.5–54.2)	23.2 (16.0-33.6)	19.0 (13.0-27.6)	0.082	**0.004**	0.915
	*p*-value	0.801	0.750	0.950			
PI [% (CI)]	PROBIOTIC	28.2 (19.1–41.6)	24.9 (16.9–36.7)	31.8 (21.7–46.7)	0.992	0.993	0.810
	PLACEBO	44.7 (30.2–66.2)	23.6 (15.9–35.1)	19.9 (13.4–29.6)	0.021	**0.002**	0.963
	*p*-value	0.274	0.997	0.262			
REC [mm (CI)]	PROBIOTIC	0.70 (0.36–1.37)	0.68 (0.35–1.33)	0.75 (0.39–1.47)	0.587	0.174	**0.048**
	PLACEBO	0.53 (0.27–1.05)	0.59 (0.30–1.16)	0.64 (0.33–1.27)	0.080	**0.002**	0.116
	*p*-value	0.571	0.765	0.749			
CAL [mm (CI)]	PROBIOTIC	2.70 (2.39–3.05)	2.39 (2.11–2.70)	2.43 (2.14–2.74)	**< 0.001**	**< 0.001**	0.845
	PLACEBO	2.69 (2.37–3.04)	2.35 (2.08–2.66)	2.31 (2.05–2.62)	**< 0.001**	**< 0.001**	0.891
	*p*-value	1.000	0.998	0.935			

Both groups showed a significant reduction in PPD between T0 and T2 (*p* < 0.01); however, no inter-group statistical significance was observed. No further significant changes in PPD were noted between T2 and T3 for either group.

BOP also showed a significant and comparable decrease over the observation period in both groups (Table 2), from 30.7% (range 21.3% – 44.1%) to 16.7% (range 11.5% – 24.2%) for the probiotic group, and from 37.9% (range 26.5% – 54.2%) to 19.0% (range 13.0% – 27.6%) for the placebo group at T3. In contrast, PI was initially lower in the probiotic group than in the placebo group, and showed a slight but non-significant increase (28.2% [range 19.1% – 41.6%] vs. 31.8% [range 21.7% – 46.7%]), while a significant decrease was observed in the placebo group (44.7% [range 30.2% – 66.2%] vs. 19.9% [range 13.4% – 29.6%]) at T3, with both groups reaching similar values.

CAL showed a statistically significant reduction between T0 and the subsequent time points, with similar reductions observed in both groups. Regarding REC, the probiotic group showed a slight decrease at T2, followed by an increase at T3. In contrast, the placebo group exhibited a progressive increase, which was significant between T0 (0.53 [range 0.27–1.05]) and T3 (0.64 [range 0.33–1.27]) (*p* < 0.01). However, the REC values remained comparable between the two groups at all time points.

**Fig. 2:**
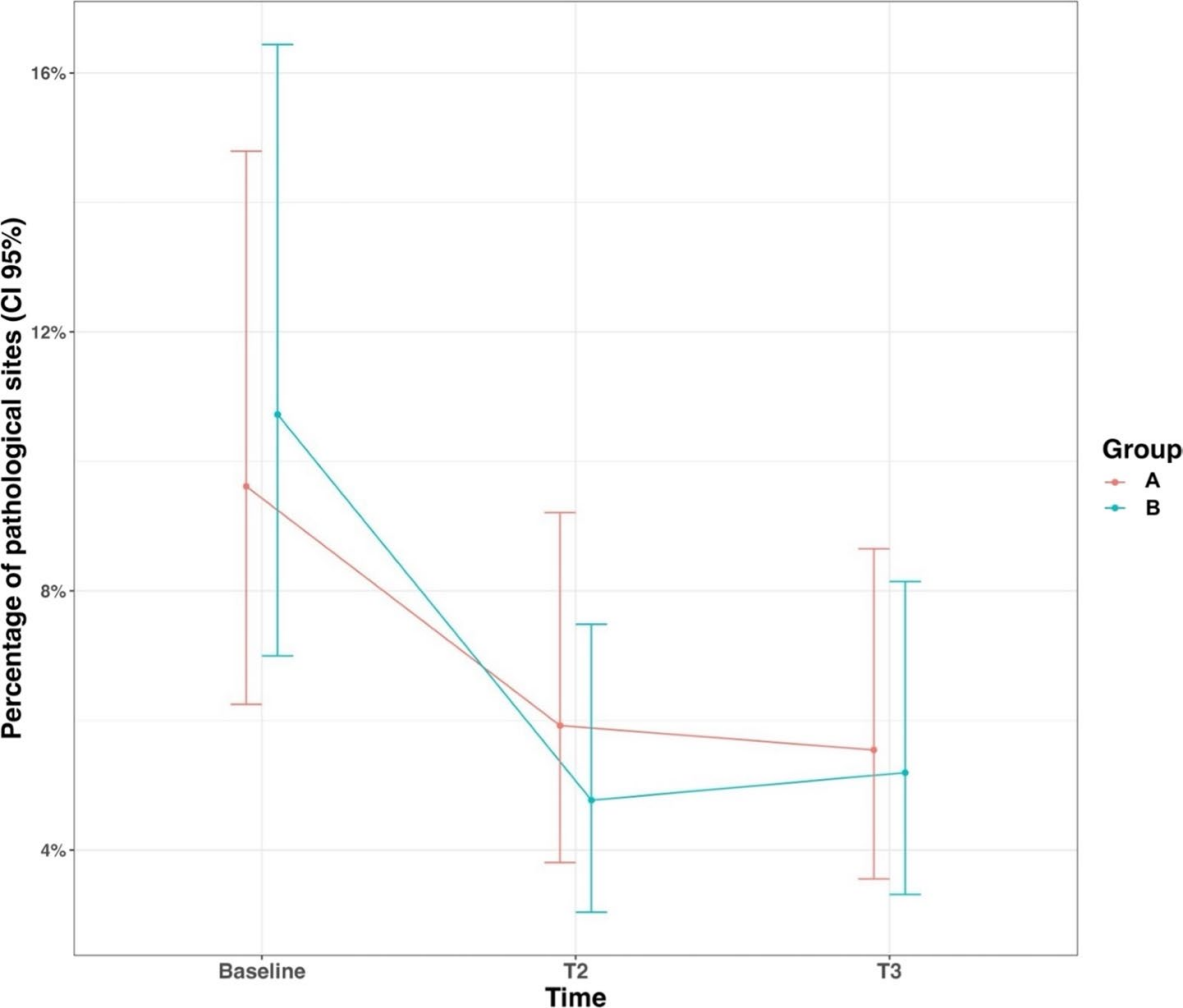
Mean percentage of pathological sites in probiotic and placebo groups at each time point. A represents the probiotic (test) group, and B represents the placebo (control) group

The percentage of pathological sites also significantly decreased for both groups from T0 to T3 (9.6% vs. 5.5% for probiotic, 10.7% vs. 5.2% for placebo (Table 3; Fig. 2)) with no significant difference between the study groups.

**Table 3:** Mean percentage of pathological sites in probiotic and control groups at each time point and inter-group and intra-group time-point comparison

Group	Baseline	T2	T3	*p*-value T0/T2	*p*-value T0/T3	*p*-value T2/T3
PROBIOTIC	9.6 (6.2–14.8)	5.9 (3.8–9.2)	5.5 (3.6–8.7)	**0.063**	**0.026**	1.000
PLACEBO	10.7 (7.0-16.4)	4.8 (3.0-7.5)	5.2 (3.3–8.1)	**< 0.001**	**0.001**	0.999
*p*-value	0.979	0.607	0.998			

**Fig. 3:**
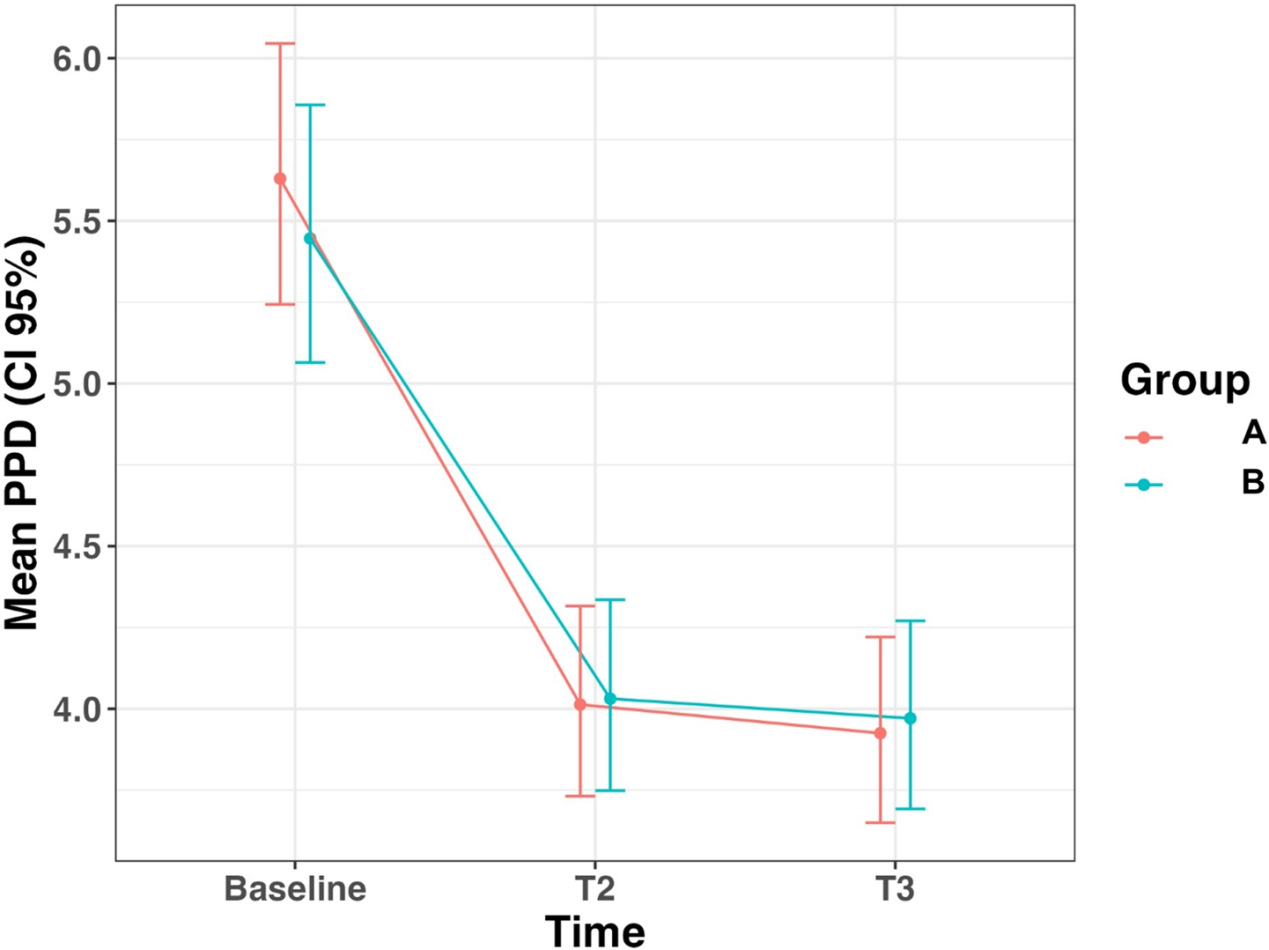
Mean PPD at pathological sites. A represents the probiotic (test) group, and B represents the placebo (control) group

When considering only the pathological sites (Table 4), PPD significantly decreased in both groups between baseline and T2/T3, with similar reductions observed across both groups (Fig. 3).

**Table 4:** Mean periodontal parameters at pathological sites at different observation times, with intra-group comparison between time points and inter-group comparison at each time point

	Group	Baseline (T0)	T2	T3	*p*-value T0/T2	*p*-value T0/T3	*p*-value T2/T3
PPD [mm (CI)]	PROBIOTIC	5.45 (5.06–5.86)	4.03 (3.75–4.33)	3.97 (3.69–4.27)	**< 0.001**	**< 0.001**	0.994
	PLACEBO	5.63 (5.24–6.05)	4.01 (3.73–4.32)	3.92 (3.65–4.22)	**< 0.001**	**< 0.001**	0.966
	*p*-value	0.890	0.999	0.994			
BOP [% (CI)]	PROBIOTIC	83.5 (76.2–88.9)	49.7 (39.5–60.0)	42.3 (32.6–52.8)	**< 0.001**	**< 0.001**	0.061
	PLACEBO	89.0 (83.3–93.0	50.9 (40.3–61.3)	38.8 (29.2–49.3)	**< 0.001**	**< 0.001**	**0.005**
	*p*-value	0.165	0.884	0.629			
PI [(% CI)]	PROBIOTIC	38.6 (25.5–51.7)	31.4 (19.3–43.6)	33.6 (21.3–45.9)	0.275	0.451	0.729
	PLACEBO	52.1 (40.2–63.9)	32.4 (18.6–46.3)	26.8 (14.0–39.6)	**0.005**	**< 0.001**	0.413
	*p*-value	0.132	0.913	0.451			
REC [mm (CI)]	PROBIOTIC	0.51 (0.21–1.27)	0.54 (0.22–1.34)	0.48 (0.20–1.20)	0.699	0.678	0.337
	PLACEBO	0.45 (0.18–1.12)	0.62 (0.25–1.55)	0.76 (0.31–1.91)	**0.085**	**0.007**	0.238
	*p*-value	0.831	0.828	0.470			
CAL [mm (CI)]	PROBIOTIC	5.79 (5.32–6.31)	4.41 (4.05–4.80)	4.41 (4.05–4.80)	**< 0.001**	**< 0.001**	0.845
	PLACEBO	5.92 (5.45–6.44)	4.28 (3.93–4.66)	4.17 (3.83–4.54)	**< 0.001**	**< 0.001**	0.891
	*p*-value	1.000	0.998	0.935			

A significant decrease was also found for BOP between baseline and T2/T3 (*p* < 0.01), with a further significant decrease for the placebo group between T2 and T3. The presence of plaque at pathological sites showed a reduction in both groups, reaching significance only for the placebo group, with no inter-group difference at any time point. REC values at pathological sites showed a slight increase in both groups at T2, which was significant only for the placebo group. At T3, the probiotic group showed a trend for REC reduction, although this reduction was not statistically significant, whilst the placebo group showed a trend for a further slight increase. The REC difference between groups was not significant at any time point. CAL reduced significantly and similarly for both groups at T2 and T3 compared to baseline.

**Fig. 4:**
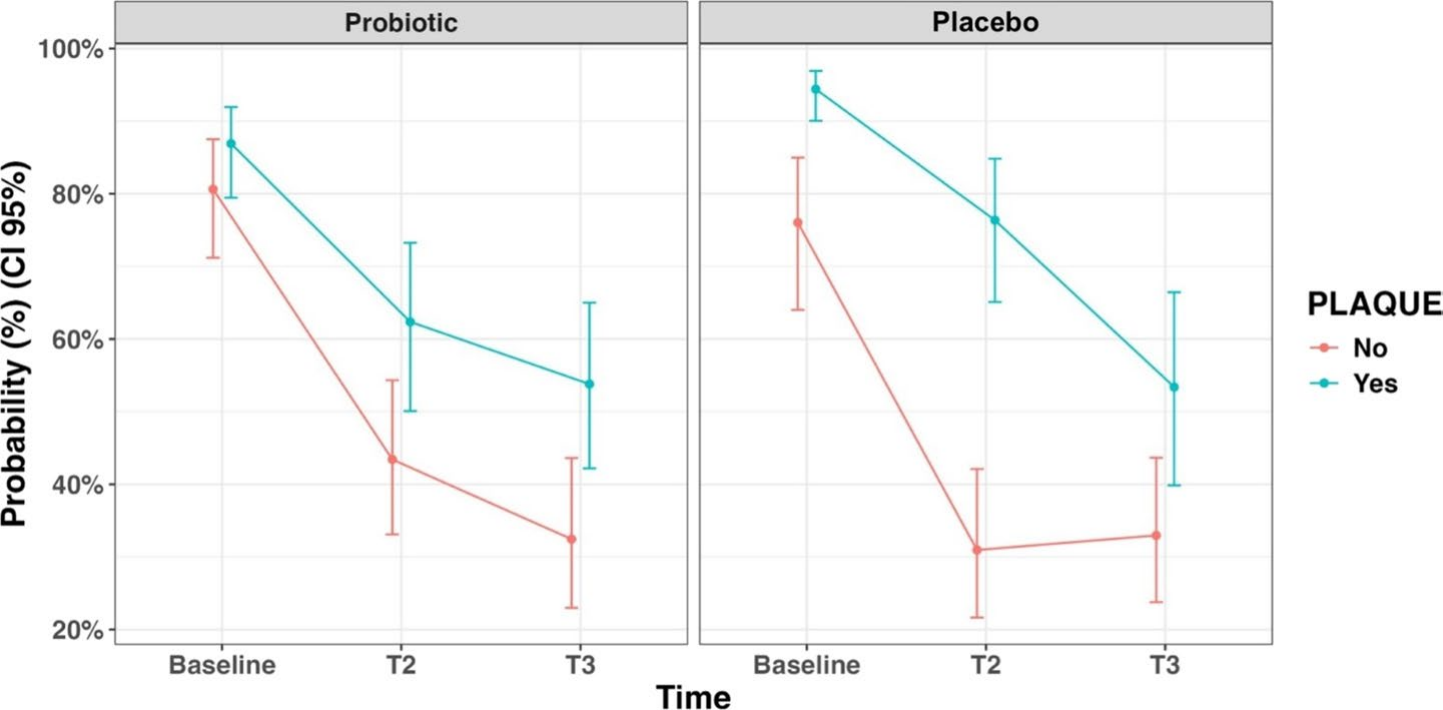
Probability that a site will be bleeding (BOP) in the presence or absence of plaque at different observation times

Finally, Fig. 4 shows the probability that a site is positive to BOP in the presence or absence of plaque at different observation times, computed from the logistic GLMM. In both groups, the BOP at pathological sites reduces post-treatment, with a more marked reduction at plaque-free sites.

### Microbiological results

A total of 209 samples were analyzed, 168 of which microbiological samples and 41 control paper points.

#### Initial sequencing data

The initial zOTU table contained 1517 zOTUs and 222 samples, including 13 sequencing controls, paperpoint controls and samples from the periodontal pockets. Since many zOTUs occurred at very low counts in only a few samples, we first removed all zOTUs with a relative abundance of less than 10^−5^, which reduced the number of zOTUs to 1289. Next, 298 zOTUs that were more abundant in the 23 “clean” (i.e. containing very few oral species) paperpoint controls than in the periodontal pocket samples were removed. The major paperpoint contaminants among the 298 removed zOTUs were identified as *Marinilactibacillus* (zOTU2, zOTU14), *Anaerobacillus isosaccharinicus* (zOTU12), *Stenotrophomonas* (zOTU16), and *Evansella* sp. (zOTU17). Some “oral” zOTUs were present in the 23 clean controls, the most abundant being: *Lactobacillales* (zOTU2), *Bacilli* (zOTU12, zOTU14), *Stenotrophomonas* (zOTU16), *Bacillaceae* (zOTU17), *Actinomuyces* (zOTU26), *Lachnospiraceae* (zOTU34), *Enterococcus sp.* (zOTU39), *Clostridiales* (zOTU45) and *Porphyromonadaceae* (zOTU46). The final zOTU table contained 991 zOTUs with 41 control paperpoint samples, 168 periodontal pocket samples, as well as 13 sequencing control samples. The subsampled zOTU table contained 11 paperpoint controls and 163 samples from the periodontal pockets.

**Fig. 5:**
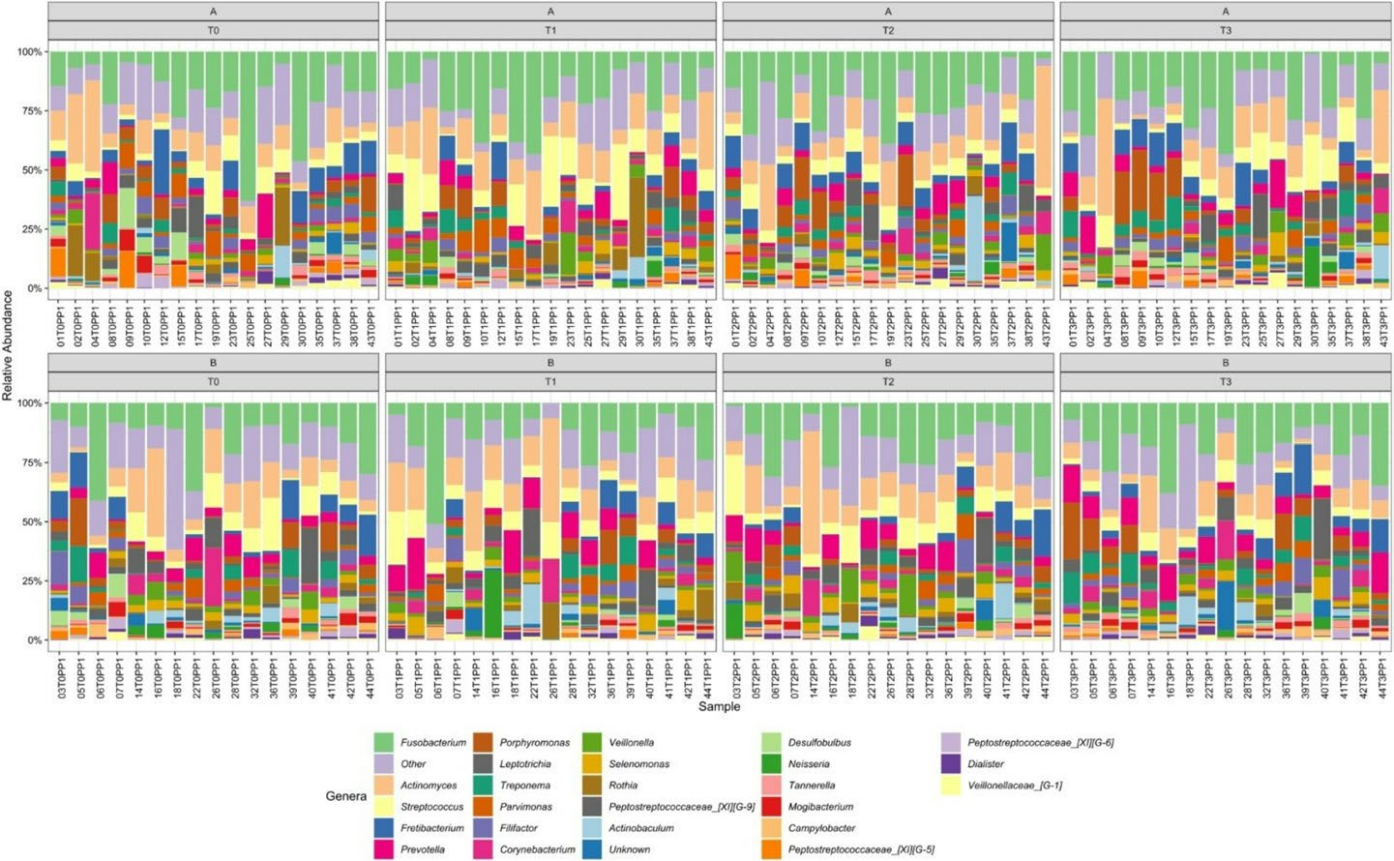
The taxonomic composition at the genus-level of 144 samples (36 subjects on all time points) per treatment group. “Unknown” refers to zOTUs which were not classified at genus level, while “Other” refers to less abundant genera (≤ 2% relative abundance, with a prevalence of ≤ 10%). A represents the probiotic (test) group, and B represents the placebo (control) group

**Fig. 6:**
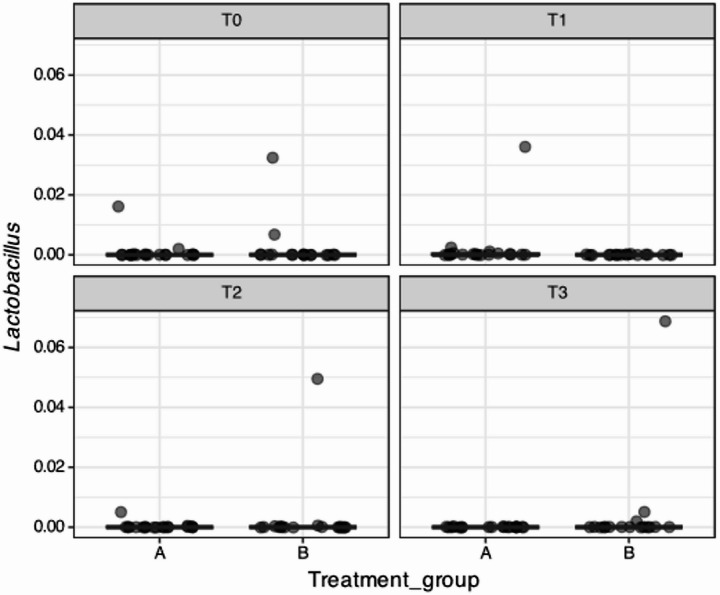
Relative abundance of genus*Lactobacillus* (including *Limosilactobacillus*) per timepoint (144 samples as in Figure 4). A represents the probiotic (test) group, and B represents the placebo (control) group

Figure 5 shows the taxonomic composition of the periodontal pocket samples per timepoint from the 36 study subjects who had data for each of the four time points, and Fig. 6 shows the relative abundance of *Lactobacillus* from the same subjects at each time point.

#### Alpha-diversity analyses

The Shannon diversity index did not differ for subjects with complete timeseries (4 time points) in the treatment groups (Friedman test; group A: 76 samples, 19 subjects, *p* = 0.27; group B: 68 samples, 17 subjects, *p* = 0.90). Cross-sectionally, no significant differences between treatment groups were observed either (Wilcoxon test).

#### Beta-diversity analyses

##### Microbial profiles and treatment groups

The differences in microbial profiles over time within the treatment groups were analyzed for each timepoint pairwise. In the placebo group only T1 vs. T3 showed a significant difference (restricted PERMANOVA, F = 1.33, *p* = 0.0006, Bonferroni corrected), whilst in the probiotic group there was a significant difference between T0 and T1 (F = 1.12, *p* = 0.038, Bonferroni corrected). Cross-sectionally, there was no significant difference between the groups at any of the four time points, not even before multiple-testing correction.

For all combinations of the time points, the within participant Bray-Curtis dissimilarity was also calculated (Suppl. Figure 1). While some small differences were present in the probiotic group, before multiple testing correction, they were mainly present because the T2 and T3 samples were more similar to each other (a lower dissimilarity).

**Fig. 7:**
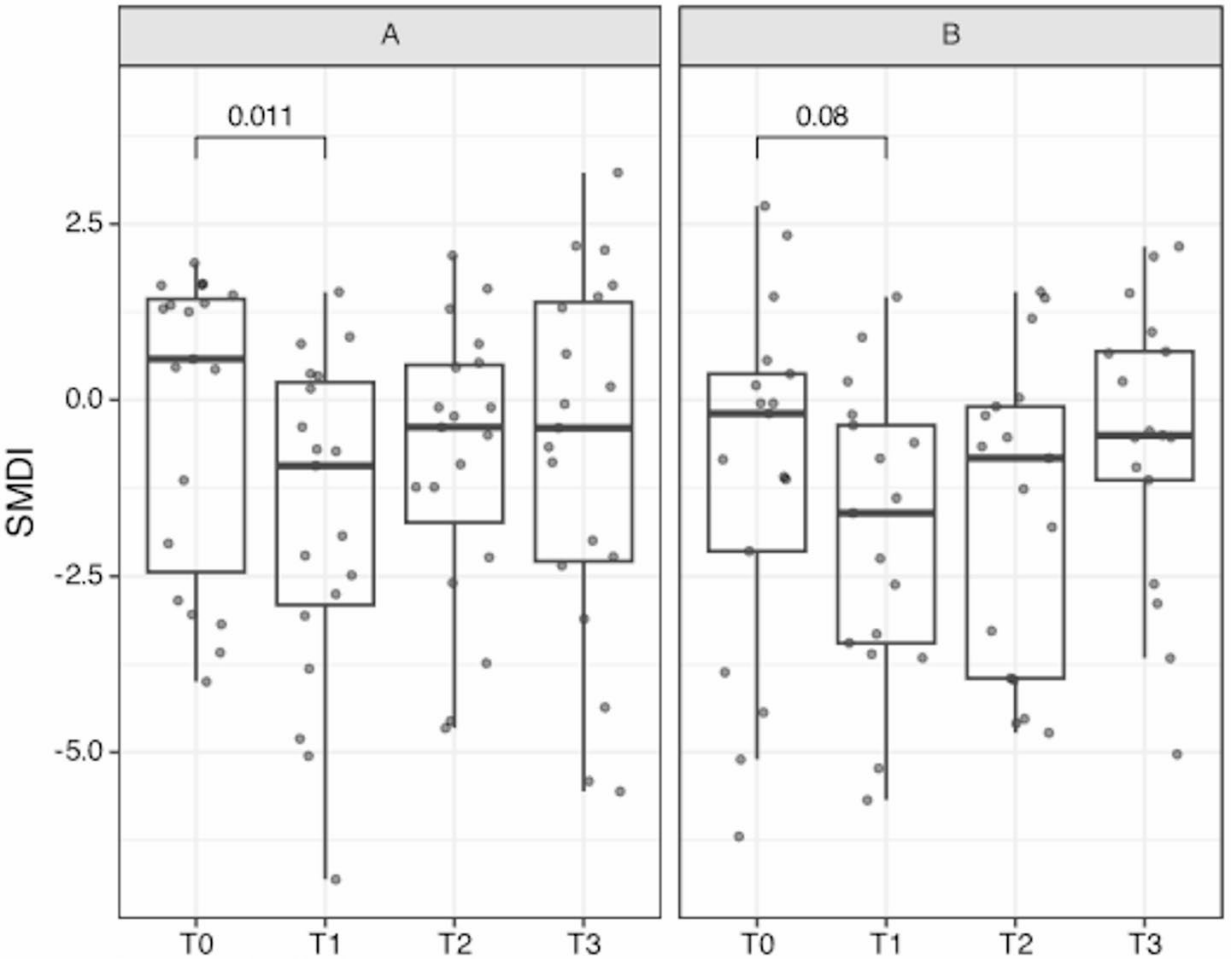
The subgingival microbial dysbiosis index (SMDI) for participants with samples at all time points. For the probiotic group (A), the at T1 a small reduction in SMDI present (before multiple-testing correction). The corresponding p-value is shown for the placebo group (B). Especially in the placebo group, the SMDI is lower at T1 and T2 compared to T3 (*p* = 5e-4, *p* = 0.02, respectively)

To further delineate possible differences in time, the subgingival microbial dysbiosis index (SMDI) [42] was calculated (Fig. 7). The SMDI is calculated using a list of normobiotic and dysbiotic species or genera, with a value around zero discriminating most of the periodontitis and healthy samples. For the V4 region used here, the values are shifted by about 1 unit [43]. A more negative value indicates a more normobiotic microbiota. Thus, when during treatment the microbiota becomes less dysbiotic, a more negative SMDI is expected.

Cross-sectionally, no significant differences in SMDI were present. However, longitudinally there was a difference (significant before multiple-testing correction) for the probiotic group from T0 to T1. In the placebo group, it also reduced from T0 to T1 (not significant), but then, as opposed to the probiotic group, was significantly increased again at T3 (T1 vs. T3: *p* = 0.003, after Bonferroni correction), indicating a return to a more dysbiotic microbial profile.

##### Differential abundance analyses

**Fig. 8:**
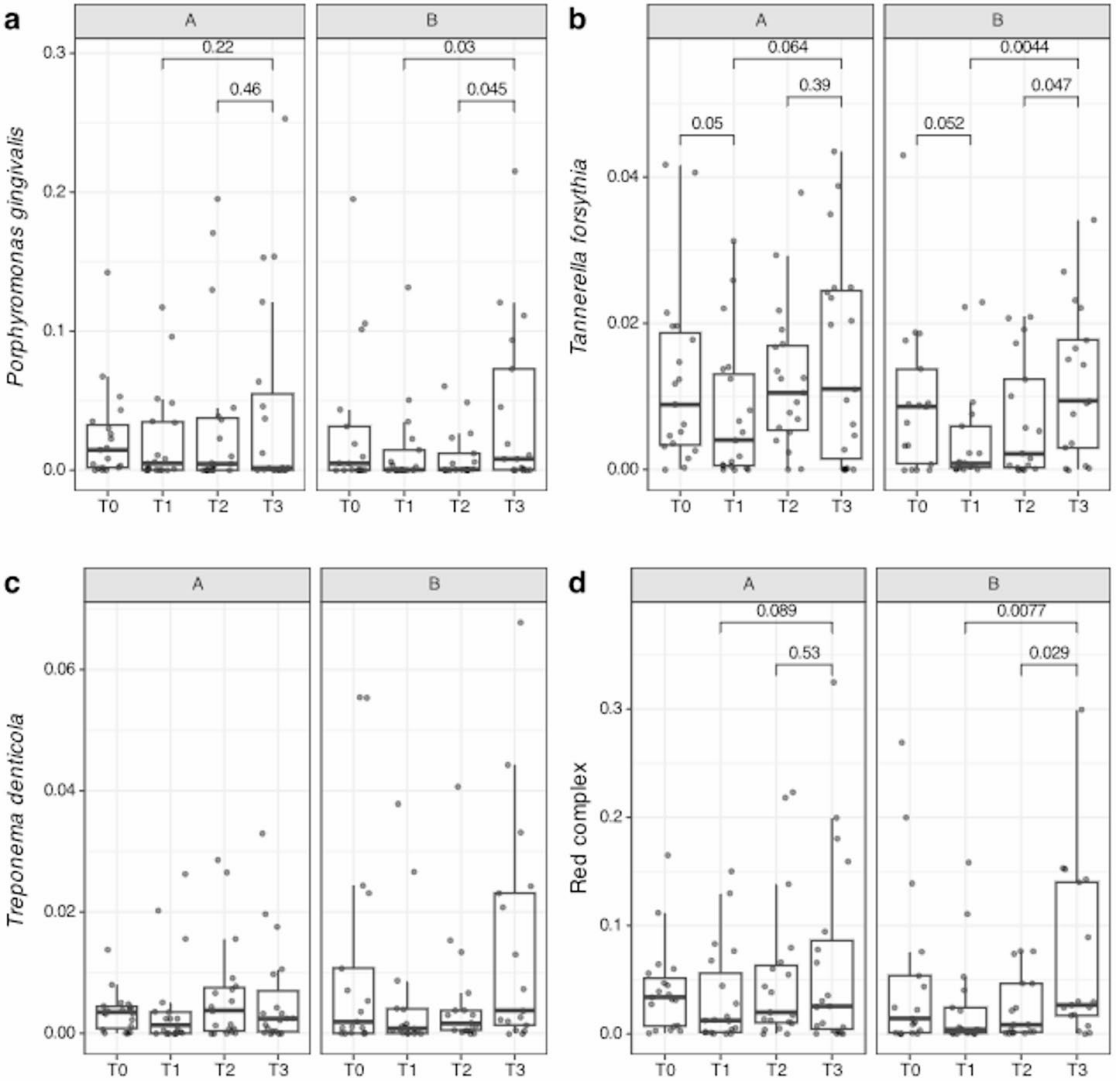
Changes in the relative abundances for the ‘red complex’ species. Participants with samples at all time points were included. Differences that were significant before multiple-testing correction are indicated with their corresponding differences in the other treatment group. Treatment A refers to the probiotic group and treatment B refers to the placebo group

Although, in general, the microbial profiles hardly differed over time and were not significantly different cross-sectionally, the relative abundances of the red complex species were analysed (Fig. 8). After multiple-testing correction, no differences remain significant. When only focusing on either the *T. forsythia* (Fig. 8b) or the combined red complex species (Fig. 8d), only a weak difference remains in the placebo group.

**Fig. 9:**
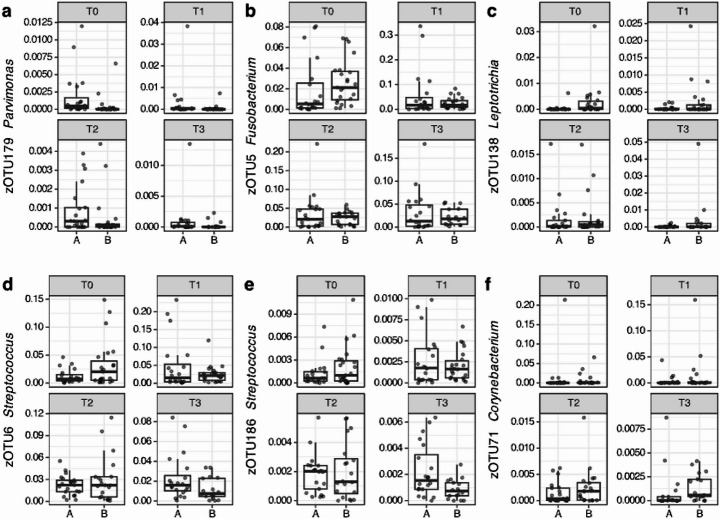
Differential abundance analyses cross-sectionally per timepoint. For a, the difference below the used thresholds was at T0 and T1, for b and c, the difference was at T0, and d, e, f at T3. For overview, all time points for these zOTUs are shown. The zOTUs were assigned the following taxonomic names at species level, respectively: zOTU179 *P. micra*; zOTU5 *F. nucleatum* subsp. *polymorphum*; zOTU138 *L. buccalis*; zOTU6 *S. dentisani*/*mitis*/*oralis*/*infantis*/*tigurinus*; zOTU186) *S. gordonii*; zOTU71) *C. durum.* Treatment A refers to the probiotic group and treatment B refers to the placebo group

Next, differential abundance analyses were performed cross-sectionally (Fig. 9), and pairwise between T0 and T1 for each treatment group (Supplementary Figs. 2 and 3). After multiple-testing correction, none of these analyses showed significantly different zOTUs or genera. However, the relatively large effect sizes could show interesting differences and zOTUs were plotted that had a significant p-value (*p* < 0.05) before multiple-testing correction and an effect-size of > |0.5|. Note that this is a mild effect size and this would not necessarily indicate statistical significance. In addition, in these figures, the relative abundance of some taxa is very low. In cross-sectional analyses at each timepoint (Fig. 9), almost only at T0 and T3, zOTUs showed a difference, using the above criteria, between the treatment groups.

#### Adherence to treatment and adverse effects

Adherence to protocol was assessed asking participants to return the empty tablet container, and a side effects questionnaire was administered. 17 patients did not fully comply with the lozenges administration schedule: 14 patients forgot to take 1–4 tablets, while 3 patients forgot to take up to a maximum of 7 tablets out of a total of 42. No adverse effects associated with the consumption of lozenges containing *L. reuteri* were reported.

## Discussion

Mechanical biofilm disruption and antibiotic/antimicrobial products remain the standard treatments for periodontal disease, but there is no proven protocol to promote the repopulation of the subgingival space with eubiotic species. Bacterial replacement therapies are a proven approach for gastro-intestinal dysbiosis, and Teughels et al. (2007) [5] were the first to test this concept in the oral environment in an animal model, applying beneficial bacteria to periodontal pockets after mechanical debridement, obtaining encouraging results. Since then, numerous studies have investigated the addition of probiotic treatments during and after periodontal therapy [11, 25–29].

The population in the present study included patients previously treated for stage III or IV and grade B or C periodontitis, with residual pockets. These patients are commonly seen in clinical practice and pose a challenge when deciding between supportive periodontal therapy (SPT) or surgical intervention. To date, no standard protocol exists for SPT with or without adjunctive therapies [46]. While several studies on the use of probiotics for oral diseases like caries and candidiasis focus on simple administration of chewing gums or lozenges, it is important to recognize that disrupting pathogenic biofilm remains a critical first step in periodontal disease treatment [27]. The same way a mature biofilm poses challenges to antibiotics/antimicrobials [47, 48], so we hypothesize that introducing beneficial bacteria would also face difficulties in establishing themselves within a mature biofilm. In line with Teughels et al. 2011 [11], our approach involved administering probiotic treatment after a session of full-mouth debridement.

The primary outcome of the present study was the reduction in PPD at test sites, which were selected based on residual PPD ≥ 6 mm or PPD = 5 mm with positive BOP at baseline (T0). Both groups showed a significant reduction in mean PPD at the test sites, with similar trends observed for CAL and REC. However, no significant differences were observed between the groups. The overall number of residual pockets at 6 months was also comparable between groups, with no difference in the full-mouth average PPD, BOP, CAL or REC. Therefore, no significant statistical differences were observed between the two groups by the end of the study, nor clinical differences that might influence the prognosis or need for further treatment.

Our PPD results contrast with those of Grusovin et al. (2020) [28] who conducted a randomized trial on the clinical and microbiological effects of a 12-week twice-daily administration of *L. reuteri* lozenges in a similar cohort of periodontal patients undergoing SPT, with GBT protocol. They observed a significantly greater reduction in PPD at 3, 6, 9, and 12 months of follow-up, as well as a decrease in BOP at 6 and 9 months in the probiotic group. The observed difference may be due to the shorter probiotic administration period in our study (3 weeks vs. 12 weeks). Moreover, Grusovin et al. (2020) [28] re-administered *L. reuteri* for an additional 12 weeks at the 6-month timepoint, which likely potentiated its effects. When comparing their 12-week data to our 3-week regimen, their results appear more promising even without considering the second course of probiotics. However, it is worth noting a recent study by Thierbach et al. (2024) [29], which found no clinically significant benefits after 3 months of continuous probiotic use in SPT patients, aside from a greater reduction in BOP. Their cohort only received one probiotic tablet per day, potentially an underdose, and lacked specific data on residual pocket parameters, making comparison to the present study and to Grusovin et al. 2020 [28] challenging.

There is currently no consensus on the timing and optimal duration of probiotic administration for periodontal treatment [26, 27]. Longer probiotic treatments are more common in cariology studies and may provide more tangible benefits. For example, Lai et al. (2021) [17] provided 60 days of probiotic therapy containing *L. brevis* to a cohort of diabetic children, noting a sustained reduction in cariogenic bacteria lasting 30 days post-treatment. In the periodontal field, re-establishment of a complex periodontal biofilm in a pocket can occur as quickly as a week after debridement [48], leading some authors to test short probiotic regimens. For example, Riccia et al. (2007) [18] observed significant reductions in gingival inflammation, plaque, calculus, and bleeding after 4 days of *L. brevis* lozenges (4 times per day). However, their study lacked initial periodontal therapy and oral hygiene control, making it difficult to assess the true effects on periodontal disease resolution. In studies investigating probiotic supplementation alongside initial periodontal therapy, 3 weeks of administration may suffice to produce significant clinical and microbiological effects. Tekce et al. 2015 [21] and Ince et al. 2015 [20] reported greater reductions in PPD and BOP up to 12 months, along with decreased inflammatory markers, in patients who received 3 weeks of probiotics. Theodoro et al. 2019 [49] did not find any additional reduction in PPD in a cohort of smoking patients but observed significant BOP reduction at 90 days. Given that Grusovin et al. (2020) [28] already tested a more prolonged administration of probiotics (12 weeks) and to try and minimise the risk of loss of compliance, the authors of the present study decided for a shorter administration time as per Tekce et al. 2015 [21] and Ince et al. 2015 [20]. A recent systematic review with network analysis by Mendonça et al. (2024) [27], published after the commencement of this study, indicated that the duration of probiotic therapy may impact its success, but found no significant benefit beyond 4 weeks of administration. It is possible that the optimal time for probiotic supplementation may coincide with initial periodontal therapy, when shorter treatments yield the greatest effects, similar to antimicrobials and antibiotics [27]. Longer supplementation, up to 3 months, and repeated supplementation might be more appropriate during SPT [28]. Further studies on SPT comparing different supplementation periods and possibly repeated administration could be of great interest, keeping in mind potential issues related to compliance and the cost-effectiveness of the intervention. Some patients in our study, despite taking probiotics for only 3 weeks, reported forgetting doses.

One interesting clinical observation from our study was that, while both groups had a high probability of BOP at T0 (around 50%) at sites with plaque, the probiotic group showed a significantly greater reduction in BOP at T2 and T3 compared to the placebo group, which exhibited only a minor decrease. A study from Demmer et al. (2008) [8] showed that the presence of periodontal pathobionts related with BOP even in sites with minimal PPD, whilst the presence of beneficial species such as *A. naeslundii* reduced the chances of BOP. However, we failed to prove any difference in the microbiological composition of sub-gingival biofilm between groups. The other hypothesis is that *L. reuteri* might have exerted a beneficial effect on the immune response of the host, through a reduction in inflammatory factors [20, 21]. Thus, supplemental administration of *L. reuteri* might be beneficial in patients with sub-optimal plaque control.

Despite no clinical differences between groups, our study highlights the importance of supportive therapy for periodontal patients, as the number of pathological pockets significantly decreased in both groups after just one session of GBT debridement. The average number of pathological sites in probiotic group decreased from 14.5 (9.4–22.2) at T0 to 8.4 (5.4–13.0) at T3, and in placebo group from 16.9 (11.0–25.8) to 8.1 (5.2–12.7), indicating a reduced need for surgical intervention [50].

Microbiological analysis from our study revealed that *L. reuteri* was detected in two patients at baseline, confirming the fact that this bacterium can be a natural colonizer of the oral cavity [51]. Some colonization of the pockets by *L. reuteri* was observed, but the rate of detection of *L. reuteri* in our test sites was much lower than that reported by Tekce et al. 2015 [21]. Nevertheless, they also could not detect any *L. reuteri* after 6 months of observation. Pocket colonization by *L. reuteri* is not often found in the literature, as the subgingival environment is not optimal for lactobacilli species [52], and permanent colonization is not expected [11, 21, 53]. Our study did not analyze supragingival biofilm or mucosal surfaces, where initial colonization might have occurred. In studies such as Teughels et al. (2013) [25], the microbiological analysis was performed in supragingival plaque, subgingival plaque and saliva samples, showing some differences. Moreover, permanent colonization is not the goal of probiotic administration. The main goal consists in the modulation of the bacterial re-colonization and of the inflammatory reaction towards a more favorable condition during the crucial post-treatment recolonization [11, 21]. Interestingly, some *L. reuteri* was found in samples from the placebo group at T2 and T3. This is possibly due to other products the patients might have used or introduced in the diet and constitutes an important factor to control in future studies.

When analyzing the overall microbial population, no significant difference was observed between the two study groups.

No significant changes were noted in the within sample diversity (alpha diversity) cross-sectionally at each time point, and between different samples (beta-diversity, PERMANOVA) at each time points between the two groups.

The SMDI is a simple index recently introduced to simplify the complexity of microbiota data sets, aiding in the determination of the level of dysbiosis of a certain microbial population, proving to be a useful tool to discriminate healthy patients from patients affected by periodonititis [42]. Its calculation not only includes health-associated taxa, such as *Actinomyces* and *Streptococcus *and all the traditionally recognized periodontal pathogens, but also more recently identified taxa, including *Filifactor*,* Mogibacterium *and *Fretibacterium *[42]. Whilst there were no statistically significant differences between groups, the probiotic group showed a significant decrease in dysbiosis from T0 to T1 (before multiple-testing correction), to return to higher levels at T3. In the placebo group, we observed a similar reduction between T0 and T1, although not significant, but also a slight increase of dysbiosis at T3 compared to baseline. The observed short-term effect in the probiotic group could be due to the probiotic administration, which was only brief and not repeated in the present study. Possibly, a longer administration [28] could have produced a more sustained effect. Another important fact to keep in mind is that oral biofilm can prove itself quite resilient to change. Factors such as saliva composition, systemic immunity and oral hygiene habits tend to keep the microbial population stable against potential perturbances such as changes in diet and administration of probiotics [54]. This would explain why the changes obtained with *Limosilactobacillus spp* seem to be only transient [21, 53]. Moreover, when the oral surfaces are fully colonised by an established biofilm, the attachment and proliferation of new bacteria can be difficult, making the biofilm mechanical disruption a fundamental step of treatment [54].

Better microbiological results might be expected when probiotic administration occurs at initial periodontal therapy. Teughels et al. (2013) [25], showed a significant reduction in *Porphyromonas gingivalis* and *Prevotella intermedia*, and Tekce et al. 2015 [21] observed a sustained reduction of anerobic species up to 180 days after probiotic administration. In the present study, at 3 months and 6 months the microbiome composition of the probiotic sites shifted back to baseline, with an increase in pathobionts such as *P. gingivalis*, *T. denticola*, *F. alocis* and *Fretibacterium*. Tekce et al. 2015 [21] observed a similar return to baseline microbiological profiles, but this occurred much later—at 1 year of follow-up. As it does not seem possible to permanently change the oral microbiome, some authors suggest repeated administration of probiotic treatment [20], but other factors, such as diet, exercise, and vitamin levels, should also be considered as they may influence long-term oral microbiome health [55–57].

The mode of probiotic administration is another important consideration. Lozenges may not be the most effective method to target residual periodontal pockets, as they require slow melting in the mouth to be effective. Teughels et al. (2007) [5] delivered a mixture of bacteria directly into the pockets, observing a significant reduction in periodontal pockets after 12 weeks. Sufaru et al. 2022 [58] applied a solution containing *L. reuteri* directly into the pockets five times over 28 days, achieving significant clinical improvement and a reduction in *A. actinomycetemcomitans*, *P. gingivalis*, and *P. intermedia*. However, Lalerman et al. 2020 [59] found no benefit from applying *L. reuteri* probiotic drops in the pockets. Local, slow-release formulations such as varnishes or gels might be worth exploring in future studies.

Further research is also needed to identify which specific bacteria or combinations are best suited to colonize the oral and subgingival environments, potentially including the newly discovered periodontal health-related oral bacteria [60]. *Streptococcus* species also seem be able to reduce the immune response to periodontal pathobionts such as *Fusobacterium nucleatum* and *Aggregatibacter actinomycetemcomitans* [61], and localised application of a mixture of *Streptococcus sanguinis*, *Streptococcus salivarius*, and *Streptococcus mitis* in periodontal pockets after root planing has demonstrated to challenge and delay the recolonisation by pathogenic bacteria and improved the bone healing in an animal model [5, 62]. It is possible that even a combination of antimicrobials and probiotics could further potentiate these effects [63]. Other strains of *Lactobacillus* have also been tested in the past. *Lactobacillus brevis* and *Lactobacillus plantarum* strains show promising clinical results (reduction of BOP and PPD), but the microbiological advantages remain unclear [64].

Finally, it is worth mentioning prebiotics. Prebiotics are nutrients of the intestinal biome, which breaks them down in short-chain fatty acids with the ability to enter the bloodstream and influence the overall organism, inducing the production of immunity molecules, increasing the population of protective species and modulating hormonal production [65]. Whilst the effect of prebiotics is widely studies in gastrointestinal conditions [65], very little literature exists on their application in patients with periodontal disease [66]. Sugar alcohols such as xylitol can be considered prebiotics in light of their ability to block acid production in the mouth and, therefore, reduce the risk of caries. However, no long-lasting shift in the oral microbiome has ever been proven [65].

One limitation of the present study is the lack of analysis of other factors that might negatively impact the chances of further healing of the residual pockets, such as smoking status and tooth type (molar vs. mono-rooted) [67]. Additionally, the absence of a standardized home oral hygiene protocol could have contributed to variability, as different oral hygiene products may influence the oral microbiome differently. Furthermore, microbiological samples were only taken from test sites, which may have overlooked shifts in the broader oral environment that could have been detected through saliva or supragingival biofilm samples.

In conclusion, the use of probiotics in periodontal patients remains a relatively novel area, and key aspects like timing, dosage, method of administration, and analysis protocols are still far from being well established or standardized. In this study, three weeks of *L. reuteri* lozenge administration in patients with a history of stage II and III periodontitis, and grade B and C, who underwent a session of supportive periodontal therapy (SPT), did not provide additional clinical or microbiological benefits when compared to SPT alone. Future studies examining longer probiotic treatment durations and alternative routes of administration would be of great interest to further explore this therapeutic avenue.

## Supplementary Information

Below is the link to the electronic supplementary material.

Supplementary Material 1

## Data Availability

Data can be shared by the authors upon reasonable request.
