# Supplementary material for: Clinical and microbiological effectiveness of limosilactobacillus reuteri in supportive periodontal therapy: randomized clinical trial
Source: Clin Oral Investig. 2025 Aug 26;29(9):422. doi: 10.1007/s00784-025-06508-w (PMC12380889; doi:10.1007/s00784-025-06508-w)
Supplement: Supplementary file 1 — Supplementary Material 1 [file 784_2025_6508_MOESM1_ESM.docx]

**SUPPLEMENTAL FIGURES**


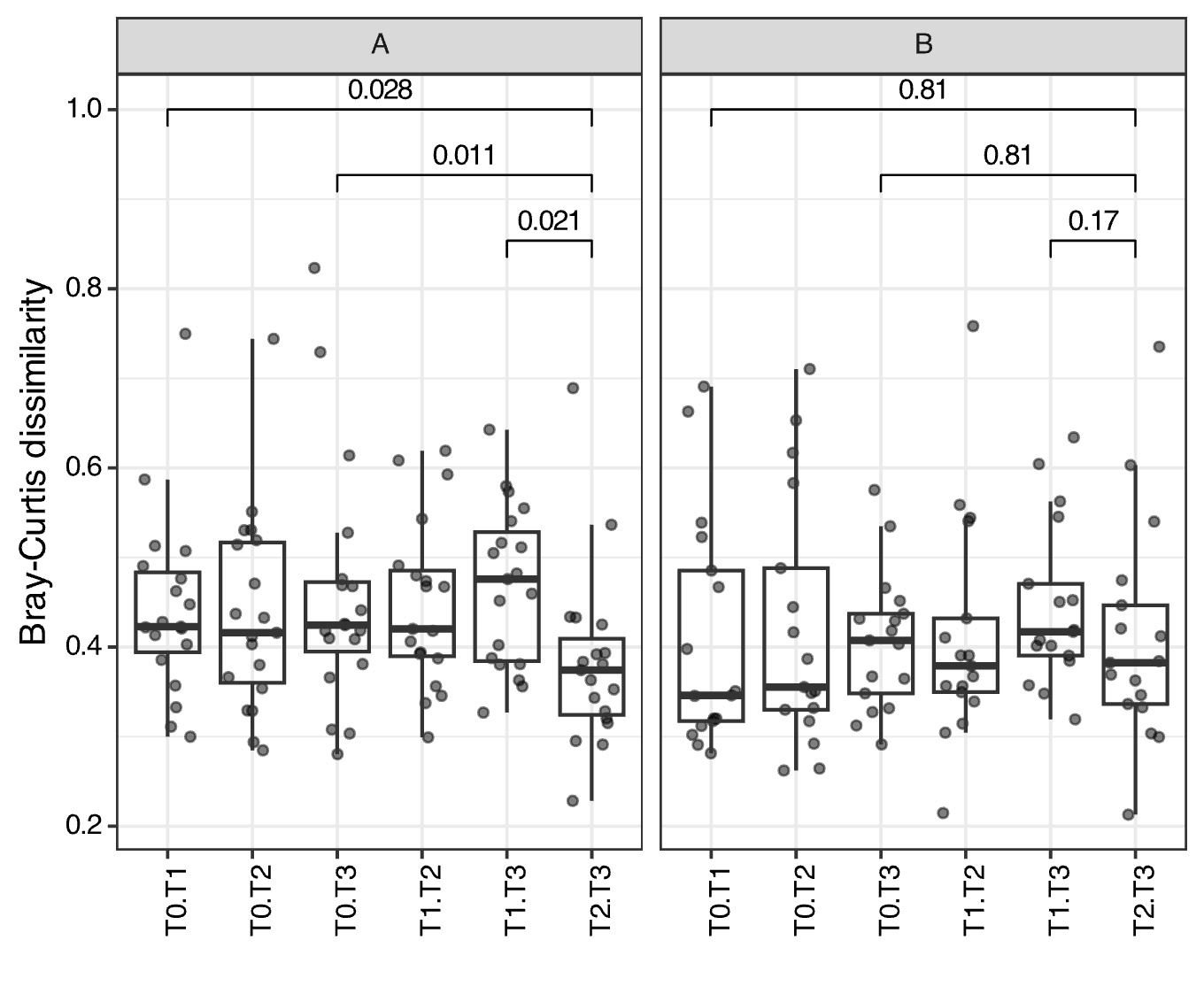


**Suppl Figure 1** - Bray-Curtis dissimilarities for the during the treatment timepoints. Differences are within the same participant. Differences, significant before multiple-testing correction, are indicated with their corresponding differences in the other treatment group. Treatment A refers to the probiotic group, and treatment B refers to the placebo group.

**
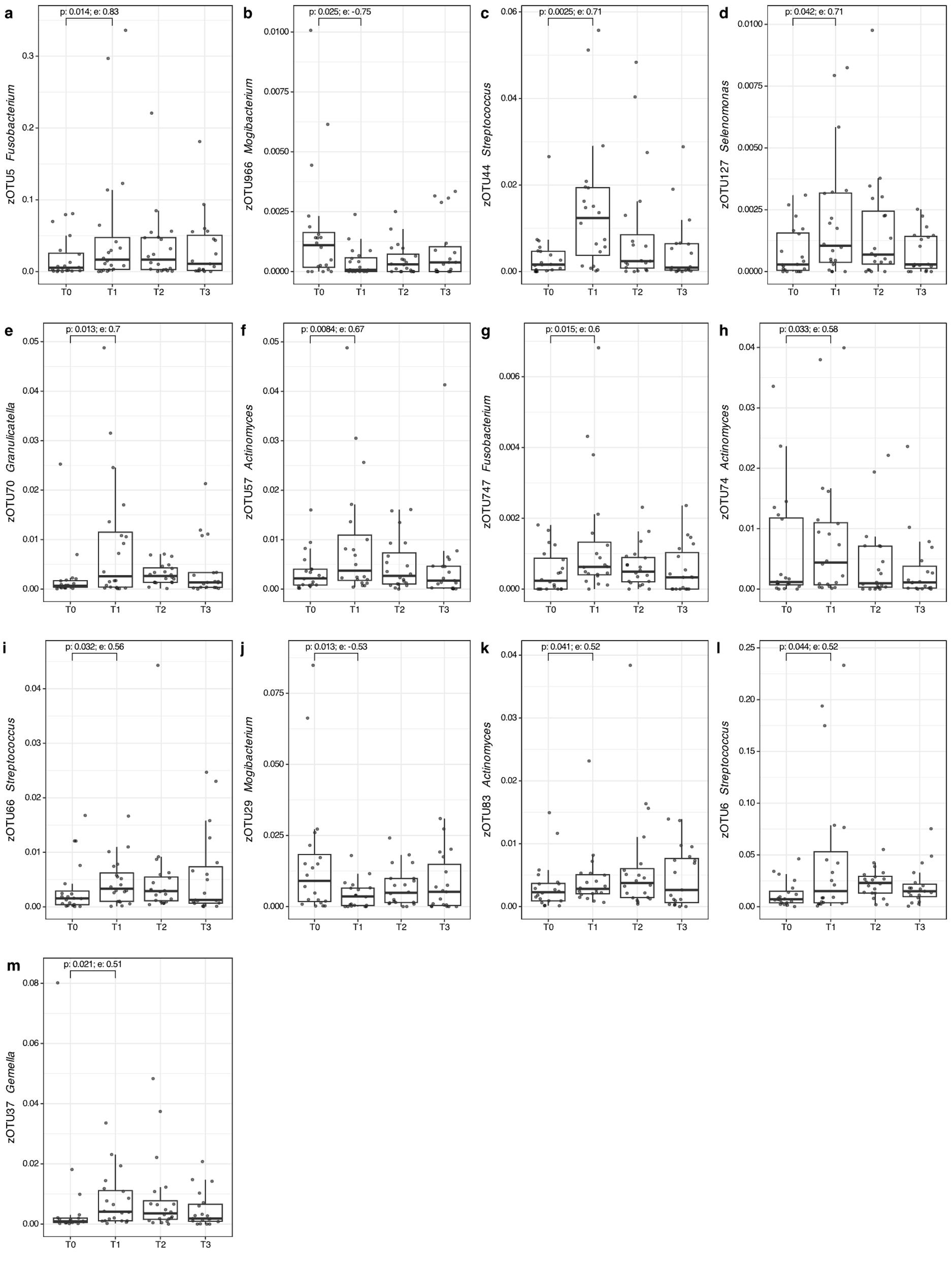
**

**Suppl Figure 2 –** ALDEx2 results for the pairwise analysis T0 and T1 for the probiotic group. For overview, all the timepoint for these zOTUs are shown. The p-value and effect size are indicated (before multiple-testing correction). The zOTUs were assigned the following taxonomic names at species level, respectively: zOTU5 *F. nucleatum* subsp. *polymorphum*, Zotu966 *Mogibacterium.*(*timidum* 98.0% id); zOTU44 *S. intermedius*; zOTU127 *S. infelix*; zOTU70 *G. adiacens*; zOTU57 *A. odontolyticus*; zOTU747 *F. nucleatum* subsp. *nucleatum/ vincentii*; zOTU74 *A. meyeri*; zOTU66 *S. gordonii*; zOTU29 *M. timidum*; zOTU83 *A. georgiae*; zOTU6 *S. dentisani/infantis/mitis/oralis/tigurinus*; zOTU37 *G. haemolysans/morbillorum/sanguinis*.

**
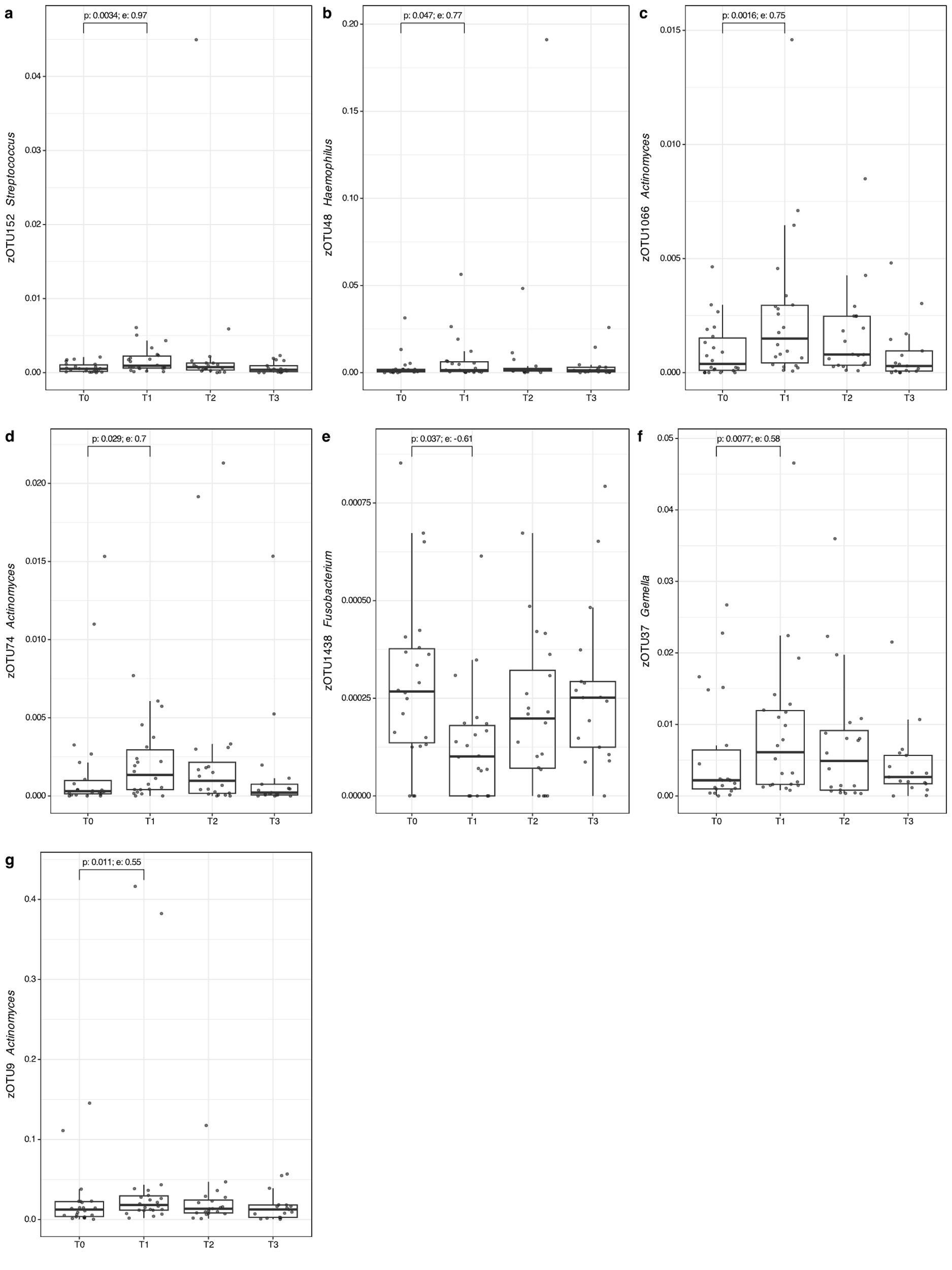
**

**Suppl Figure 3 –** ALDEx2 results for the pairwise analysis T0 and T1 for the placebo group. For overview, all the timepoint for these zOTUs are shown. The p-value and effect size are indicated (before multiple-testing correction). The zOTUs were assigned the following taxonomic names at species level, respectively: zOTU152 *S. australis/parasanguinis_I/parasanguinis_II*; zOTU48 *H. parainfluenzae*; zOTU1066 *Actinomyces* (*johnsonii/naeslundii* 98.0% id); zOTU *A. meyeri*; zOTU1438 *Fusobacterium* (*nucleatum subsp._polymorphum* 97.2% id); zOTU37 *G. haemolysans/morbillorum/sanguinis*; zOTU 9 *A. naeslundii/oris*.
